# Comparing lying behaviour of young riding horses on days in an individual indoor box, on an outdoor paddock alone, or in pairs and in the following night

**DOI:** 10.1111/evj.14041

**Published:** 2024-01-01

**Authors:** Pia Helmerich, Iris Bachmann, Lorenz Gygax

**Affiliations:** ^1^ Animal Husbandry & Ethology, Faculty of Life Sciences, Albrecht Daniel Thaer‐Institute of Agricultural and Horticultural Sciences, Humboldt‐Universität zu Berlin Berlin Germany; ^2^ Agroscope, Haras National Suisse HNS Avenches Switzerland

**Keywords:** horse, REM sleep, security, sleep deprivation, social context

## Abstract

**Background:**

Horses must lie down to go into vital rapid‐eye movement (REM) sleep. If they are not lying down for sufficiently long periods they can become so sleep‐deprived that they collapse uncontrollably, which results in a risk of injuries.

**Objectives:**

To investigate how recumbency as a prerequisite to REM sleep on the experimental days and the following nights was influenced by changes in social and spatial environment throughout the day.

**Study design:**

Cross‐over design in which subjects experienced each experimental condition twice.

**Methods:**

Observations were conducted on a horse farm. Ten young horses in training were observed on days when they were alone indoors in a box, on an outdoor paddock alone, on the same paddock in pairs, and in the following night stabled alone. The number of lying bouts and the total lying duration throughout the day and night were assessed automatically using 3D‐accelerometers and data were evaluated using mixed‐models.

**Results:**

Horses had a higher number of lying bouts during the days (*p* = 0.05, by a factor of 1.21 [95% CI: 1.00–1.45]) and longer lying duration at night (*p* < 0.001, by a factor of 11.25 [6.47–18.40]). On average, the number of lying bouts and the duration of lying increased from being indoors alone, to being outdoors alone, and outdoors in pairs although this could not be statistically supported (bouts: *p* = 0.5, by a factor of 1.08 [0.84–1.36] and 1.17 [0.91–1.48]; *p* = 0.6, duration: by a factor of 1.39 [0.73–2.93] and 1.38 [0.68–2.78]).

**Main limitations:**

A small number of horses were observed and there was large variability between days within horses.

**Conclusions:**

We found some indications that open space and a social companion throughout the day increased time lying down in the day as well as during the following nights thus allowing for more REM sleep.

## 1 INTRODUCTION

Apart from feed intake, resting takes up an important proportion of a horse's time budget.^1^ Horses are extreme flight animals and, accordingly, a major part of resting is performed while standing,^2^, ^3^ even deep sleep (slow‐wave‐sleep^3^, ^4^). Yet, even horses can enter a state of complete relaxation necessary for survival only during rapid‐eye movement (REM) sleep^5^, ^6^ and do so solely for a short time.^7^ Because muscles are relaxed in this sleep phase, this type of sleep can be performed only while lying down. Not lying down, which has been related to horses perceiving they are not in a sufficiently secure environment,^3^, ^6^ can lead to equine health problems. Horses can collapse uncontrollably if tiredness increases over time (sleep deprivation)^8^ and it is important for horse welfare that conditions allow sufficient amount of time for REM sleep.

Previous studies have found that companion horses allowing for distribution of vigilance among several herd members increases the lying time.^9^ Moreover, horses prefer a soft lying surface for lying^10^, ^11^, ^12^ with sufficient space.^13^, ^14^ Inappropriate barn structure or a low rank of a horse within a herd reduces the amount of time that horses lie down.^15^, ^16^ Lying behaviour as a pre‐requisite to perform REM sleep has not been studied previously in young horses trained to be ridden. This is a potentially difficult phase for the horses as they are confronted with intensified human contact, many new procedures and situations, need to exert themselves more and rest may be specifically important. In the current study, we investigated lying behaviour in horses spending daytime alone in an indoor box, alone on an outdoor paddock, or in pairs on the same paddock and during the following night when housed individually in the adjacent indoor boxes independent of the daytime condition. We expected that a visually open environment and a social partner would increase lying behaviour as reflected by the frequency of lying bouts and the total duration of lying.

## 2 MATERIALS AND METHODS

### 2.1 Animals and housing

The study was conducted on a farm boarding and training horses (Stahnsdorf, state of Brandenburg, Germany). We recruited 10 young warmbloods (6 mares and 4 geldings; 4–9 years old) with withers height > 148 cm that were used to be kept in pairs formed from the 15 young horses on the farm. All horses were healthy and none had stereotypic or other abnormal repetitive behaviours. All these horses were being prepared as riding horses and were worked 5 days a week. Each horse had 2 days off per week, usually spent in outdoor paddocks. These days and the following nights were used for our observations.

During the night, horses were stabled individually in 1 of 10 boxes situated on both sides of a passage (2 m wide) and that measured 3 × 4 m (12 m^2^) with two windows to the outdoors (1 m^2^ each). The partitions between the boxes and towards the passage consisted of metal bars up to a height of 2.4 m above a 1.3 m high tiled wall. All horses had unrestricted visual and olfactory contact with the other horses. The boxes were littered with straw and cleaned daily. Water was available ad libitum from automatic drinkers. Hay was provided three times daily (at 06:30, 16:00 and 21:30) and concentrate feed twice daily (oats and/or pellets at 07:00 and 17:00). In front of the building, six paddocks (16 × 16 m) with sand on the ground were arranged in two rows on both sides of a riding arena (20 × 60 m). They were surrounded by wooden fences with beams at 0.5 and 1.4 m of height and additional fence tapes at 1 and 1.40 m of height. Hay was available in freestanding racks and was replenished at noon. Water was available ad libitum from plastic self‐drinking basins.

### 2.2 Experimental design

The measurements took place between early March to late May 2022. The animals were kept individually in adjacent indoor boxes over night between 17:00 and 07:30 h of the following day. In the daytime between 08:30 and 16:00 h, the horses went through three different experimental conditions. The remainder of the time (16:00 to 17:00 and 7:30 to 08:30 h) was used to relocate the horses. The three conditions were that the horse spent the day either individually in an indoor box (‘box’), alone in an outdoor paddock (‘alone’), or with a companion horse in the outdoor paddock (‘in pairs’). All horses had been habituated to the three conditions previously in regular management procedures. Conditions used on the days between our observations were determined by farm managers. The pairs of horses were familiar with each other and the same pairs were always in the paddock together. Horses studied outdoors were there for the full day while when studied in boxes, horses were taken into the boxes by 11:00 h at the latest.

We used two repetitions for each horse. In each repetition, a horse was exposed to the three experimental conditions (2 repetitions × 3 conditions × 10 horses = 60 24‐h periods) with different sequences of the conditions in the two repetitions. Accordingly, we had to choose 20 sequences (10 horses × 2 repetitions) for the experimental conditions. Six different sequences were possible given the three experimental conditions. These six possible sequences were distributed across the 20 sequences needed in such a way that each possible sequence was used at least three times (and two sequences were used four times).

### 2.3 Data collection

Lying behaviour was automatically recorded using 3D‐accelerometers (MSR‐logger type 145 W, MSR Electronics GmbH) that have been used for horses before.^12^, ^17^ The acceleration recorded with 1 Hz was used on the vertical leg direction (Y‐axis). The loggers were foam padded and attached to the left foreleg at the height of the cannon bone using a horse bandage (according to guideline by Hess and colleagues^18^) and secured with tape (Tesa Duct Tape 4610, Tesa SE).

The csv‐files exported from the loggers were further evaluated using an R script. First, for each acceleration value on the Y‐axis, it was decided whether it was lower than −0.75 (standing) or higher (lying). This value was then smoothed using a running median across 31 values corresponding to a time window of 31 s. That is, a majority ‘vote’ across these 31 values was taken to assign ‘lying’ or ‘standing’ to each 1 Hz measurement.

The time between 08:30 and 16:00 h was considered for the daily, the time between 17:00 and 07:30 h for the nightly measurements. Because the durations of the daily and the nightly measurements were different, the number of lying bouts and the total lying duration were calculated as per hour of observation for comparability.

### 2.4 Data analysis

Statistical analysis was conducted using Bayesian linear mixed‐effects models in R (Version 4.2.0^19^) based on the default non‐informative priors as implemented in the package blme.^20^ The log‐transformed number of lying bouts [per h] and duration of lying [h per h] served as outcome variables in one model each. The fixed effects were the experimental condition (factor with three levels: box, alone, in pairs), the time of day (factor with two levels: day, night) and their interaction. Horse identity crossed with calendar date served as random effects to account for the dependency within repeated measurements of the horses and measurements of different horses taken during the same 24 h period (based on the default unstructured positive definite variance–covariance matrix). Model assumptions, that is, normality of the residuals and homogeneity of variance, were checked using a graphical analysis of residuals (package DHARMa^21^) and no major deviations were apparent. *p*‐Values were calculated based on a parametric bootstrap (PBmodcomp; package pbkrtest^22^). Parametric bootstrap was also used for the calculation of confidence intervals.

## 3 RESULTS

All horses lay down at least once in every night. During the day, one horse did not lie down in one repetition of each condition, one horse did not lie down in one repetition of box and in pairs, and two horses did not lie down in one repetition of box and alone, respectively.

**FIGURE 1:**
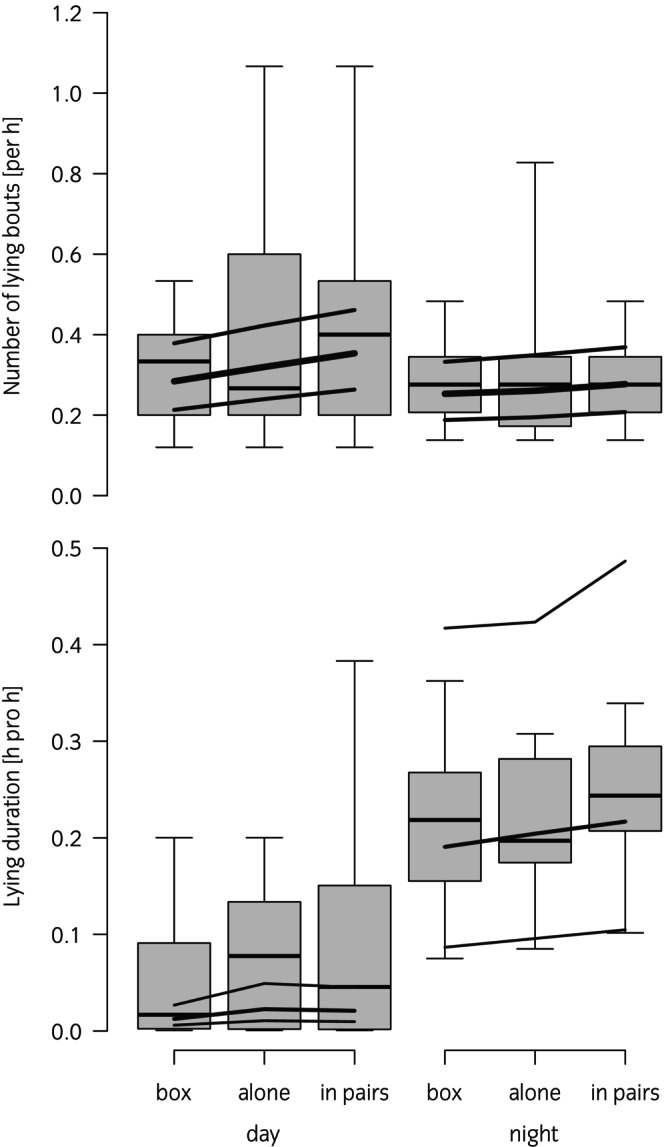
The number of lying bouts (top) and the lying duration (bottom) in young horses (*n* = 10) experiencing three experimental conditions during the day: in an indoor box; in paddocks alone and in paddocks in pairs with data from the following night. Note that all horses were housed individually overnight regardless of daytime experimental conditions. Boxplots show the median, the quartiles and the range of the raw data. Thick lines: model estimate, thin lines: 95% confidence interval.

The number of lying bouts increased on average from box to alone and in pairs during the day as well as the night but this pattern could not be supported statistically (global test: *p* = 0.5; main effect condition: *p* = 0.5; interaction: *p* = 0.8). The number of lying bouts was higher during the day than the night (main effect time of day: *p* = 0.05; Figure 1, top).

The lying duration also increased from box to alone and in pairs on average during the day as well as the night but this pattern could not be supported statistically, either (global test: *p* = 0.05; main effect condition: *p* = 0.6; interaction: *p* = 0.7). The lying duration was higher during the night compared with the day (main effect time of day: *p* < 0.0001; Figure 1, bottom).

## 4 DISCUSSION

The young horses in training investigated here had more and shorter lying bouts throughout the day and longer total lying times in the night, which was not surprising. The majority of resting in horses takes place at night.^23^ Disturbances from within and from outside of the enclosures that are more common may lead to interrupted lying bouts increasing their number throughout the day.^24^ Overall, the young horses observed here lay around twice as long in the box compared with previous data,^15^ which potentially may be explained by the younger age. This is supported by the fact that foals lie again about twice as long as the young horses in our study.^25^

We did not perform a formal power analysis for our sample size because little was known about the variability in lying behaviour within and between individual horses for such young animals. Given that our main focus (the different experimental conditions) was a within‐subject effect, we reached a sample size with sufficient residual degrees of freedom to estimate the model stably. This allowed for the interpretation of average effects even if p‐values might remain high due to the large variability in the data. In future studies, the inclusion of sex, the exact age, and the effect of the other individual in a pair are of interest. Because these were between‐subject effects in our study, the number of animals (*n* = 10) were insufficient for a systematic investigation of these questions. The overall differences between the individual horses were accounted for in our analysis based on the random effect of horse included in our statistical evaluations.

On average, the horses had an increase in the number of lying bouts and longer total lying durations (which implies relatively constant durations of the single lying bouts) from in the box individually, to singly outdoors and outdoors in pairs. In future studies, this difference may be supported more easily if longer phases across several days with constant treatments were provided. Throughout the day, several reasons may account for this pattern. Differences between the indoor box and the outdoor paddock may be explained by different materials on the ground although straw may be the preferred lying material compared with sand,^3^, ^11^, ^12^ the increased space available,^3^, ^13^, ^14^, ^26^ or the open view in the paddock might have provided a sense of security.^3^, ^7^ The two conditions outdoors differed in the social component, which again implies a heightened sense of security in pairs that led to more lying overall. It is rather surprising that this pattern was still visible at night, when conditions were constant, the horses being alone in their boxes. Here, no direct differences between the three conditions can account for the pattern, and it is possible that the sense of security perceived throughout the day may have been carried over into the following night. This would mean that the horses felt more secure in the night following a day with a higher sense of security, lying for longer and potentially allowing a higher amount of REM sleep. This would be in spite of the fact that some social security was provided in the boxes because neighbouring horses could be seen and heard. Given the high variability in the data within the horses (and the correspondingly high *p*‐values for the difference of the conditions), this sense of security appears to be a minor contribution to the lying time in any given night for these young horses in training. Another contributor may have been the weather.^27^ Other factors^3^ that influence horse sleep such as light, environmental temperature and the resulting core body temperature, and exercise were kept as constant as possible in our study within the repetitions of each horse given our within‐subject study design. Still, it seems that providing the potential for vigilance^7^ and social contact^1^ promotes lying to some extent in young horses during training and this may be clinically important because providing these situations may lead to a decreased risk of extreme tiredness in the horses and a decreased risk of injury due to horses collapsing from fatigue.

## FUNDING INFORMATION

Not applicable.

## CONFLICT OF INTEREST STATEMENT

The authors have declared no competing interests.

## AUTHOR CONTRIBUTIONS

All authors were involved in designing the study, Pia Helmerich collected the data and wrote a first draft of the manuscript and all authors finalised and approved the draft.

## DATA INTEGRITY STATEMENT

Lorenz Gygax had access to all data and takes responsibility for data integrity and data analysis.

## ETHICAL ANIMAL RESEARCH

Ethical committee oversight not required for this non‐invasive study.

## INFORMED CONSENT

Owners of horses were orally informed of the study content and research question and gave their consent.

### PEER REVIEW

The peer review history for this article is available at https://www.webofscience.com/api/gateway/wos/peer-review/10.1111/evj.14041.

## Data Availability

The data that support the findings of this study and the R evaluation code are available at https://doi.org/10.17605/OSF.IO/WTQ4D.
